# Peeling mechanism of tomato induced by HHAIB: Microscopic, ultrastructure, chemical, physical and mechanical properties perspectives

**DOI:** 10.1016/j.fochx.2023.101028

**Published:** 2023-11-22

**Authors:** Yu-Hao Zhou, Sriram K. Vidyarthi, Parag Prakash Sutar, Buer Ha, Qing-Hui Wang, Fa-Tao He, Ming-Qiang Xu, Wen-Qiang Zhang, Hong-Wei Xiao

**Affiliations:** a College of Engineering, China Agricultural University, P.O. Box 194, 17 Qinghua Donglu, Beijing 100083, China; b Institute of Agro-products Storage and Processing, Xinjiang Academy of Agricultural Sciences, Urumqi, Xinjiang, China; c School of Food and Strategic Reserves, Henan University of Technology, Zhengzhou 450001, China; d Department of Biological and Agricultural Engineering, University of California, Davis, One Shields Avenue, Davis, CA 95616, USA; e Department of Food Process Engineering, National Institute of Technology Rourkela, Odisha 769008, India; f Agricultural Mechanization Institute, Xinjiang Academy of Agricultural Sciences, Urumqi 830091, China; g Jinan Fruit Research Institute, All China Federation of Supply and Marketing Coorperatives, Ji'nan 250014, China

**Keywords:** Peeling, Microstructure, Ultrastructure, Water state, Pectin contents, Young's Modulu

## Abstract

In order to better manage the peeling degree and avoid unnecessary losses, the current work aimed to explore the peeling mechanism of a novel peeling technology, high-humidity hot air impingement blanching (HHAIB). The relationships between HHAIB peeling performance and the changes in skin temperature, skin structure, water state, pectin fractions content, and skin mechanical properties of tomatoes were analyzed. Results showed, after HHAIB treatment, the epicuticular wax was disrupted, the skin exhibited more and longer random cracks, the degradation of inner skin tissue was observed by transmission electron microscopy, the free water percentage increased resulting in water loss in the whole tomato, the water-soluble pectin contents decreased in tomato fleshes, while the contents of chelate-soluble pectin and sodium-carbonate-soluble pectin increased. HHAIB heating reduced the elongation at break, and increased Young's Modulus of tomato peel. This study revealed the HHAIB peeling mechanism and provided new insights for developing HHAIB peeling technology.

## Highlights

• The mechanism of peeling tomatoes using HHAIB technology was investigated.
• Peel cracking and loosening was observed from tomato epidermal tissues treated by HHAIB heating.
• SEM, LSCM and TEM observation explained crack behaviors of tomato skin.
• The water state and cell-wall polysaccharides changes of tomato fleshes explained peeling loosening.
• HHAIB treatment increased the Young's Modulus of tomato skin making more likely to crack.

## 1 Introduction

With high moisture content and perishable tissue, fresh tomato is difficult to preserve and its shelf life is short. So, it is often processed in various foods such as tomato juice, tomato sauce, tomato powder, canned tomato and dried tomato products. Peeling is an essential unit operation before processing of tomato into canned or dried whole cherry tomato products since peeling can improve palatability, enhance osmotic dehydration or drying rate, and decrease pesticide residue (Vidyarthi et al., 2019a, Zhou et al., 2022b). The conventional peeling methods include hot lye and hot water or pressurized steam peeling due to easy operation and mass production (Garcia & Barrett, 2006). However, lye peeling usually results in a large amount of wastewater containing high salinity, chemical oxygen demand, and organic solids, which may cause considerable negative impacts on the environment (Wang et al., 2014). In addition, hot water peeling also has adverse effects on the environment due to the discharge of a large quantity of wastewater containing soluble nutrient substances (Deng et al., 2019b, Zhou et al., 2022b). Developing sustainable and non-chemical novel peeling technologies tempts tomato processors to eliminate chemical contamination and wastewater generation (Wang et al., 2014). For the first time, Zhou et al. (2022a) employed the high-humidity hot air impingement blanching (HHAIB) technique for tomato peeling, and determined that the optimal condition was 110 °C heating temperature in combination with 40 % of relative humidity and 75 s treatment time. HHAIB heating, using high humidity hot air impingement on the material surface, is more efficient than traditional superheated steam heating (Deng et al., 2019b, Wang et al., 2023). HHAIB peeling technique can avoid wastewater discharge and effectively reduce chemical residue and the loss of water-soluble nutrients while significantly improving peeled tomatoes' texture and nutritional quality compared to lye peeling and hot water peeling (Zhou et al., 2022a). However, as a novel thermal peeling technique, the mechanism of peeling performance change induced by HHAIB peeling is still unknown.

From the point view of industrial processing, it is essential to understand the peeling mechanism to control better the degree of peeling, thus avoiding unnecessary losses (Ayvaz et al., 2016). Peel loosening and peel cracking are two critical steps for the successful separation of flesh and peel, which involves thermally induced physicochemical changes of tomato exocarp and flesh (Vidyarthi et al., 2019b). The tomato exocarp consists of an external epidermal layer covered with the waxy cuticle and two to four layers of collenchymatous cells. The flesh beneath the exocarp tissue is mainly composed of parenchyma and collenchyma (Mintz-Oron et al., 2008). The waxy cuticle's structural property and the tomato skin's mechanical performance are prominent in peel cracking (Hetzroni, Vana & Mizrach, 2011). For example, Floros and Chinnan (1990) found that the waxy layer of skin could significantly hinder the mass transfer because the diffusion rate of sodium hydroxide in water was almost 103 times than that through tomato's peel. Thus heating was needed to degrade the wax layer and crack the peel. Wang et al. (2014) characterized the mechanical properties of tomato peel during infrared peeling heating and found that the viscoelastic behavior of tomato peel was closely related to the peeling properties. Li et al. (2014a) also observed that infrared radiation heating increased Young's Modulus of tomato peels and made tomato skin easy to crack. In addition, several possible mechanisms were proposed that the breakdown of pectic substances of hypodermal cells and vaporization of the cell fluids caused peel loosening during thermal peeling (Li et al., 2014a, Shen et al., 2020, Gao et al., 2018). However, these results and conclusions are speculative, and the exploration of the peeling mechanism is limited, lacking reliable structural evidence about the change of the pectic substances and the water distribution as well as water state for the peeling process.

Therefore, the objectives of the current work were to explore HHAIB peeling mechanism of tomato. The changes in skin temperature, skin cracks, microstructure and ultrastructure of epidermal tissues, water state, the content of pectin polysaccharides, and skin mechanical properties during HHAIB peeling were analyzed. This study aimed to study the relationship between micro-level cell and structural change and macro-level HHAIB peeling performance change, and explain the HHAIB thermally induced physical and biochemical changes of tomato peel and flesh that are associated with peel loosening and cracking behavior, as well as provide some theoretical basis for the study of peeling mechanism.

## 2 Materials and methods

### 2.1 Materials

Cherry tomatoes (*Solanum Lycopersicum* var. *Cerasiforme*) with a uniform red ripening stage were purchased from a local supermarket (Beijing, China). Defect-free tomatoes were collected and stored at 4 ± 1 °C until use within 3 d from purchase. Before processing, raw fruit were equilibrated at room temperature.

### 2.2 Peeling method and peeling performance evaluation

The HHAIB equipment and peeling procedure were previously described in detail by Wang et al. (2017) and Zhou et al. (2022a). The HHAIB processing experiments were performed under an optimized condition, i.e., air velocity of 14.0 ± 0.5 m/s, processing temperature of 110 °C, relative humidity of 40 %, and processing time of 75 s (Zhou et al., 2022a). The tomatoes were treated for different durations (15, 30, 45, 60, and 75 s) to explore the HHAIB peeling mechanism. The surface and center temperature changes of tomatoes were monitored by using thermocouples (type-K, ETA Sensors Technology, China). Fresh tomatoes treated by manual peeling were used as a control group.

### 2.3 Confocal laser scanning microscopy (CLSM) observation

The surface structure of tomato skin treated by different HHAIB times was observed using confocal laser scanning microscopy (CLSM) (Sansofar, Spain). Three-dimensional imaging of tomato peel (5 mm × 5 mm) was performed to evaluate the extent of skin rupture and cuticle loss. The sample was observed using a laser scanning confocal objective (*EPI* 20) with 20x magnification (Zhang et al., 2020).

### 2.4 Microstructure and ultrastructure observation

#### 2.4.1 Microstructure by scanning electron microscope (SEM)

Small cubes (1 cm × 1 cm × 0.5 cm) of tomato with the skin attached and pieces (1 cm × 1 cm) of tomato peel were taken from the equatorial region of the tomato fruit. The samples were coated with a thin layer of gold film to observe the microstructure by SEM (SU3500, Hitachi High-Technologies Corporation, Tokyo, Japan) with an accelerating voltage of 15 kV (Li et al., 2014a, Zhang et al., 2023).

#### 2.4.2 Ultrastructure by transmission electron microscopy (TEM)

TEM (Hitachi High-Technologies Corporation, Tokyo, Japan) was used to visualize the cell ultrastructure of tomato skin. Samples with size of 3 mm × 2 mm from different HHAIB pretreated tomato skin were prepared for TEM detection following the methodology described by Deng et al. (2018). The samples were fixed with 25 mL/L glutaraldehyde fixative solution and 40 g/L paraformaldehyde fixative solution in 0.1 mol/L sodium phosphate buffer (pH 7.2) for 2 h. After three washes of 15 min with the buffer, the samples were post-fixed in10 g/L osmium tetroxide fixative solution in the same buffer for 2 h. After that, three washes using phosphate buffer were performed. All fixed tissues were dehydrated in a graded series of ethanol. Following dehydration, the tissues were filtrated and transferred to propylene oxide and then to propylene oxide and Spurr's resin mixture. Then, the tissues were immersed in pure Spurr resin overnight at 4 °C and embedded in Spurr resin. Finally, for TEM analysis, the blocks were cut by Leica EM UC6 ultramicrotome (Leica Microsystems, Wetzlar, Germany).

### 2.5 Measurements of water states with low field nuclear magnetic resonance (LF-NMR)

The relaxation time determinations of fresh and HHAIB-treated whole tomatoes were performed using an LF-NMR analyzer (Niumag, Suzhou, Jiangsu. China) according to the methods described by Wang et al. (2018). Carr-Purcell-Meiboon-Gill (CPMG) sequence was employed to measure the spin–spin relaxation time (T_2_) and percentage of the water distribution (A_2_). The primary sampling parameters were set as follows, sampling bandwidth (SW) = 100 KHz, time waiting (TW) = 3000 ms, time echo (TE) = 0.8 ms, and number of sampling (NS) = 8.

### 2.6 Pectin extraction and determination

#### 2.6.1 Preparation of alcohol insoluble residue (AIR)

The AIR of tomato was prepared as described by Deng et al. (2019b). The freeze-dried sample was transferred into 100 mL 95 % (v/v) boiling ethanol for 15 min, and then the residue was filtered. The above procedure was repeated thrice. Then, the residue was washed with acetone and washed three times. The final residue was collected and dried overnight at 40 °C as AIR.

#### 2.6.2 Pectin fractionation and pectin content

Pectin is the main component of the middle lamella that binds adjacent cells and acts as a plasticizer in the cell walls between the cellulose/hemicellulose network. Therefore, the hypodermal tissue (thickness is about 5 mm) attached to the removed peels was collected and its pectin content was determined.The separation of pectin fractions was conducted according to the method described by Redgwell, Melton, & Brasch (1992) and Deng et al. (2019b). Distilled water, 0.05 M cyclohexane- *trans*-1, 2-diamine tetra-acetic acid (CDTA, pH 6.5) containing 0.1 M potassium acetate, and 0.05 M Na_2_CO_3_ containing 0.02 M NaBH_4_ were used to obtain water-soluble pectin (WSP), chelator-soluble pectin (CSP), and sodium-carbonate-soluble pectin (NSP). The galacturonic acid (GalA) contents of the three kinds of pectin were analyzed by the colorimetric hydroxyl-phenyl-phenol method (Deng et al., 2019b). The absorbance was monitored at 530 nm, and results were expressed as mg GalA/g AIR. Analyses were performed in triplicate.

### 2.7 Determination of the mechanical property of the tomato skin

The mechanical properties of tomato peels were determined according to the method of Vidyarthi et al. (2019b). The tomato peel from the equatorial region in the longitudinal direction was cut into a rectangular segment (3 cm × 1 cm). Both ends of the peel segment were clamped with the DTG tensile fixture. The tensile attributes of peel were investigated by a texture analyzer (CT3, Brookfield Ltd., USA) with an initial clamping distance of 1 cm, a trigger force of 25 g, and a stretching rate of 5 mm/s until it failed. The force–deformation curve (Fig. 7(a)) and peak force were recorded and the maximum force was identified as the rupture force. The elongation at break and Young's modulus were calculated by the method of Hetzroni, Vana, and Mizrach (2011). Five replicates for each peeling treatment condition were evaluated.

### 2.8 Statistical analyses

Statistical significance was analyzed by one-way ANOVA analysis (Duncan's test) using the SPSS statistics software (Version 25.0, SPSS Inc., Chicago, IL, USA). The differences were considered statistically significant at p < 0.05 throughout the study.

## 3 Results and discussion

### 3.1 Peeling performance and surface temperature changes

**Fig. 1:**
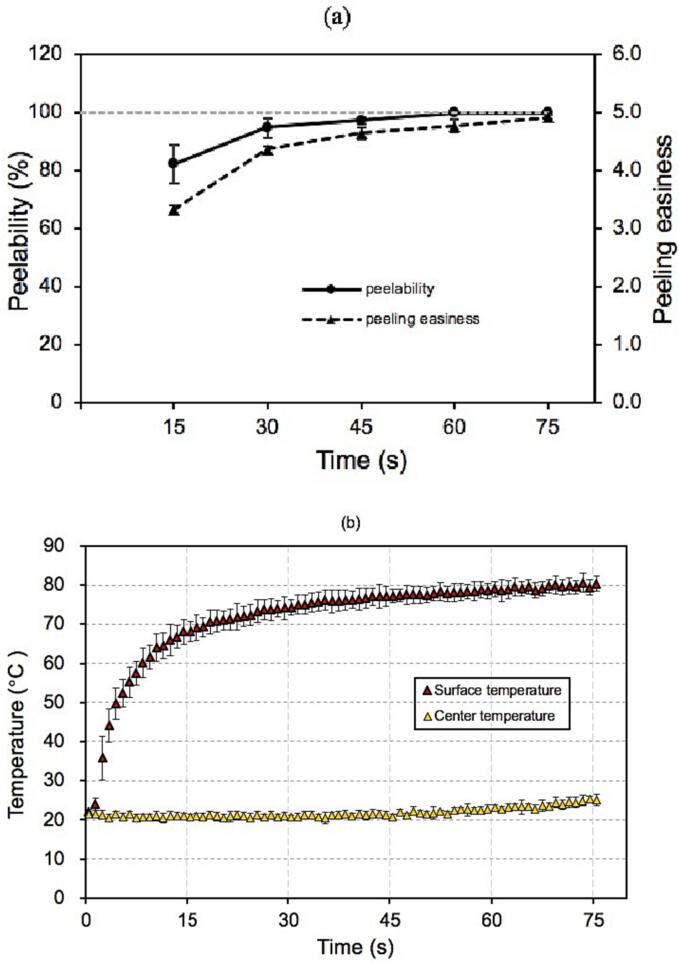
The evolution of the relative activity of peroxidase and polyphenol oxidase (a) and the surface and the centre temperature changes during HHIAB heating (b).

In general, as HHAIB processing time increased, the peelability and peeling easiness increased, which resulted from heat transfer in the exocarp area of tomatoes. The optimized HHAIB peeling parameter was determined to be 110 °C heating temperature in combination with 40 % of relative humidity and 75 s of treatment time according to the previous study by Zhou et al. (2022a), as shown in Fig. 1(a), and the time when the peeling rate reaches 100 % and the peeling easiness reaches more than 4.7 was defined as the processing endpoint (Zhou et al., 2022a). Under this optimized condition, the surface temperature of tomatoes increased markedly from room temperature to above 80 °C. The centre temperature of tomatoes did not change significantly, as shown in Fig. 1(b). Similarly, Li et al. (2014a) studied the temperature profile of the infrared radiation heated tomatoes and found that the surface temperature of tomatoes was about 80 °C when reaching the peeling endpoint. As a thermal treatment technique, material temperature is the most direct factor affecting the HHAIB peeling performance. Still, adequate thermal energy that caused physical and biochemical changes in tomato skin and flesh is considered to be the leading underlying causes of significant changes in peelability and peeling easiness. Based on the proposed peeling mechanism reported by previous studies (Li et al., 2014a, Shen et al., 2020, Gao et al., 2018), the samples treated by different HHAIB times, 15, 30, 45, 60, and 75 s, were chosen as objectives for the subsequent experiments to monitor the changes in the microstructure of tomato skin, the ultrastructure of skin cells, moisture state, mechanical properties of tomato skin, and pectin contents during heating, to thoroughly explain the HHAIB peeling mechanism.

### 3.2 Microstructure changes of tomato skin and ultrastructure changes of skin cells

**Fig. 2:**
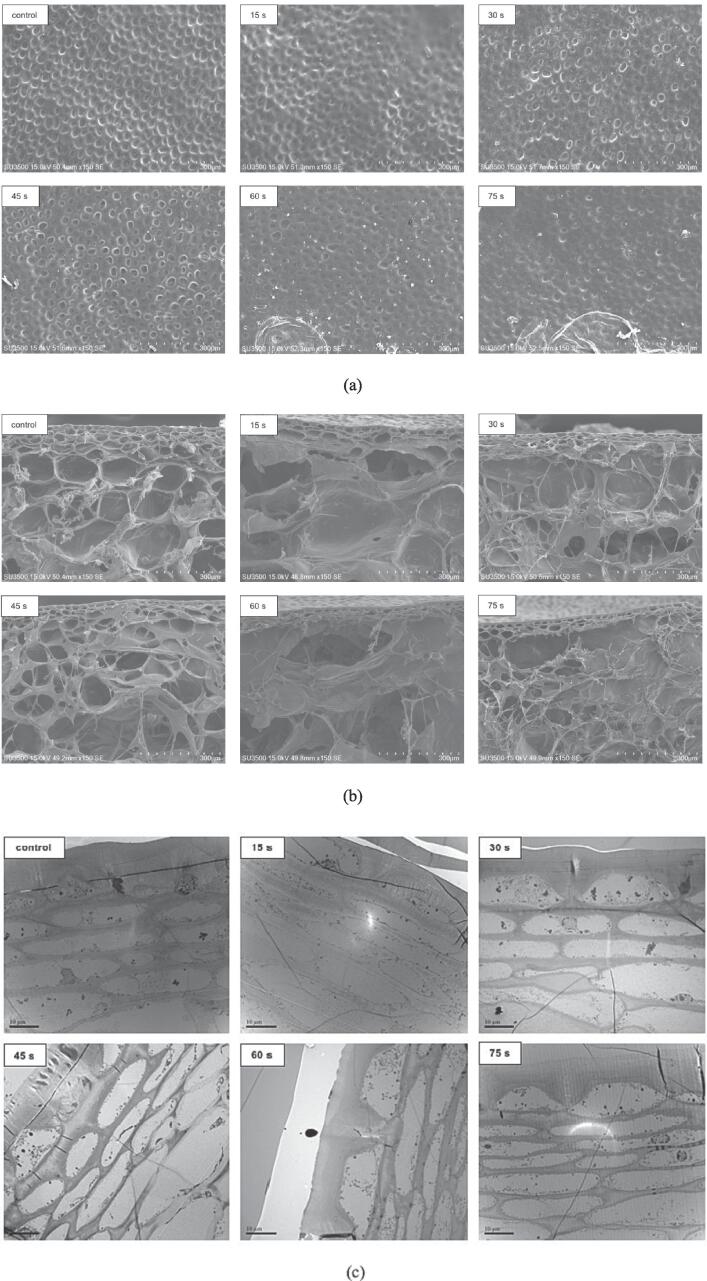

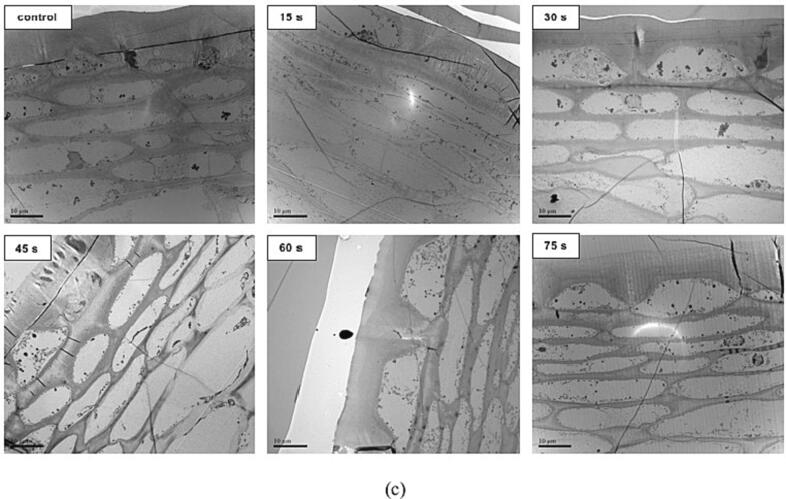
Scanning electron microscopic images of outermost tomato surface (a) and cross-sectional images of the pericarp (b), and the ultrastructural changes of transverse sections of tomato skin (c) during HHAIB heating.

The microstructure changes on the tomato skin surface for both fresh and HHAIB-treated tomatoes were observed by SEM, as shown in Fig. 2(a). On the fresh tomato skin surface (control), clearly defined and intact contours of cell wall structures could be observed. In the case of the samples treated by HHAIB, the overall shape and contour became blurred and the number of micro-cracking increased gradually with the increase of HHAIB time. Additionally, circular shrinkage was observed for the samples after 60 and 75 s of HHAIB heating, among which the circular shrinkage of the sample treated with 75 s of HHAIB processing was more significant. Previous studies speculated that the above phenomenon regarding the changes in epidermal structure was mainly caused by the phase transition of the waxy layer and structural damage on the cuticular membrane because HHAIB has a higher heat transfer rate compared to traditional superheated steam heating, causing overheating damage of tomato skin within a very short processing time (Zhou et al., 2022a).

**Fig. 3:**
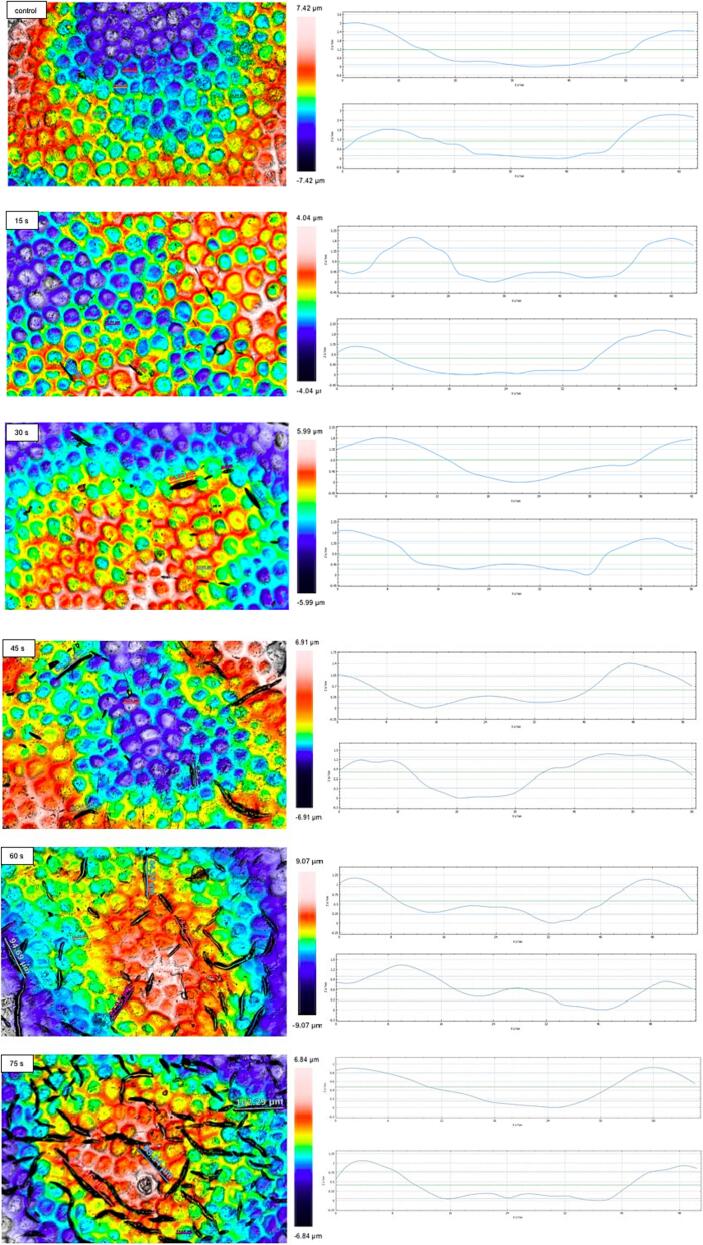
LSCM images of tomato skin surface during HHAIB heating, with the height difference on the tomato skin surface (blue coloured line). (For interpretation of the references to colour in this figure legend, the reader is referred to the web version of this article.)

To validate this speculation, higher resolution and more detailed LSCM images were provided; it quantitatively confirmed the micro-cracking and waxy cuticle change. As shown in Fig. 3, the structure of the epidermal cells in fresh tomato skin (control) is polygonal, the central area is concave, and the contour part is higher than the epidermal level, which is in agreement with the research of Gao et al. (2018) and Fava et al. (2017). Fig. 3 illustrates the epidermal structure changes of HHAIB-treated tomatoes with different heating times. It was found that the epidermal layer exhibited more and longer random cracks, and the length of the cracks increased from about 39 μm to about 103 μm when the HHAIB heating time increased from 15 to 75 s. In addition, LSCM images showed that HHAIB treatment significantly altered the height difference between contour and internal depression of epidermal cells. With the increase in the HHAIB heating time, the height difference reduced from about 3.0 in fresh tomatoes to about 2.0, 1.8, 1.4, 1.3, and 1.0 in HHAIB treated samples for 15, 30, 45, 60, and 75 s, respectively. These findings confirmed that HHAIB led to the melting and redistribution of the epicuticular wax layer, thus resulting in a less apparent epidermal cell contour. In contrast, the contour shape of epidermal cells had a slight change only.

The structure of the outer pericarp tissues is complex, and the LSCM images showed clear morphology on the surface of tomato skin, but the deep structural changes were not observed. Therefore, TEM was used for observing the transverse sections of tomato skin to obtain more detailed views (Fig. 2(c)). The tomato dermal system consists of a cuticular membrane, one single layer of epidermal cells, and a two to four cell thick layer of hypodermal cells (Mintz-Oron et al., 2008, Li et al., 2014a). Epidermal cells with a quadrangular shape have a thick outer tangential wall and thinner inner wall, and a conspicuous wedge is exhibited between two consecutive epidermal cells (Fava et al., 2017). The outer wall of an epidermal cell contains thick continuous layers, which comprise four defined layers from outer to inner, including the epicuticular waxy layer, the cuticular membrane (constituted by only cuticle proper and cutinized layer mainly composed of intracuticular wax, cutin, and polysaccharide matrix), the digestive layer composed of pectic polysaccharides, and the non-cuticular cellulose layer (Fava et al., 2017). As shown in Fig. 2(c), the fresh sample (control) showed that the outer tangential epidermal cell wall had a smooth and clear contour; the structure of epidermal cells and hypodermal cells was intact with clear organelles; and the hypodermal cells were cytological integral with regular cell shape, uniform cell wall thickness, well-defined middle lamella and intact cell membrane, which was similar to those observed by Fava et al. (2017). After blanching for 15 s, only slight plasmolysis and loose organelle distribution were observed in epidermal and subcutaneous cells. With the increase of blanching time, the thickness of the outer wall of the epidermal cell gradually decreased, the degree of structural damage on the exterior wall gradually increased, and the outline of epidermal cells gradually became smooth from irregular quadrilateral and the cell volume increased slightly. Meanwhile, it was also found that the hypodermal cells gradually deformed, and the cell wall of hypodermal cells became more swollen and damaged with the increased blanching time. It showed that HHAIB heating caused the alteration of the organization of skin extracellular cuticles and the degradation of skin inner tissue, thus promoting peel cracking.

### 3.3 Microstructure changes of cross-section

A cross-section of the outer pericarp tissue and the flesh for fresh and HHAIB heated tomatoes are shown in Fig. 2(b). Beneath the thick-walled subcutaneous cells in the outer pericarp tissue are the larger and rounder parenchymatous cells in the mesocarp (Li et al., 2014b, Liu et al., 2020). The precise cell shapes, intact cell wall contours, and highly structured arrangement of cells could be observed in the control sample. After HHAIB heating, it was found that the phenomenon of layer separation between skin and mesocarp was more and more evident with the increase of heating time. Upon treatment for 15 s, the parenchymatous cells in the uppermost layer of the mesocarp were partially broken, and the parenchymatous cell contours in the lower layer were slightly blurred, but the complete cell structure could be observed. With the increase in treatment time, the parenchymatous cells adjacent to the hypodermal cell layer were ruptured seriously, and the fissures between the hypodermal cell layers in the exocarp and parenchymatous cell layers in the mesocarp gradually enlarged. Especially with an HHAIB treatment time of 75 s, the skin cell layers were almost separated from the mesocarp cell layers, which indicated that the thermal effect of HHAIB significantly disrupted the skin layer and adjacent parenchymatous cells, which resulted in subsequent layer separation.

The degree of layer separation was significantly correlated with peeling easiness. The layer separation between the skin layer and parenchymatous cell layers in the mesocarp could be due to the differences in the size and wall thickness of the cells in different tissue types (Mohr, 1990, Li et al., 2014a). As shown in Fig. 2(b), tomato cell size increased from smaller skin cells to larger parenchymatous cells. The tissues with smaller cells tend to have a greater content of cell walls and lower intercellular space volume, making the tissue firmer. This abrupt cell size gradient resulted in the difference in mechanical properties of cells exposed to heat and thereby contributed to the tendency for layer separation (Li et al., 2014a, Toivonen and Brummell, 2008). The previous reports of Mohr (1990) and Li et al. (2014a) also showed that the difference in cell structural characteristics of tomatoes contributed to layer separation during the tomato peeling process and the ease of peeling.

In addition to differences in cell structure, the reason for the phenomenon of layer separation induced by HHAIB heating was assumed to be related to other multi-physical and biochemical phenomena, including mechanical failure of the cell due to an increase in the internal vapor pressure caused by cell fluid vaporization, and cell-wall polysaccharide degradation caused by thermal damage between the skin and parenchymatous cells (Floros and Chinnan, 1988, Garrote et al., 2000). To substantiate these explanations, the change of water state of a whole tomato and the change of cell wall polysaccharides of tomato flesh during HHAIB heating were explored further and are reported in the following section.

### 3.4 Water states and fractions changes

**Fig. 4:**
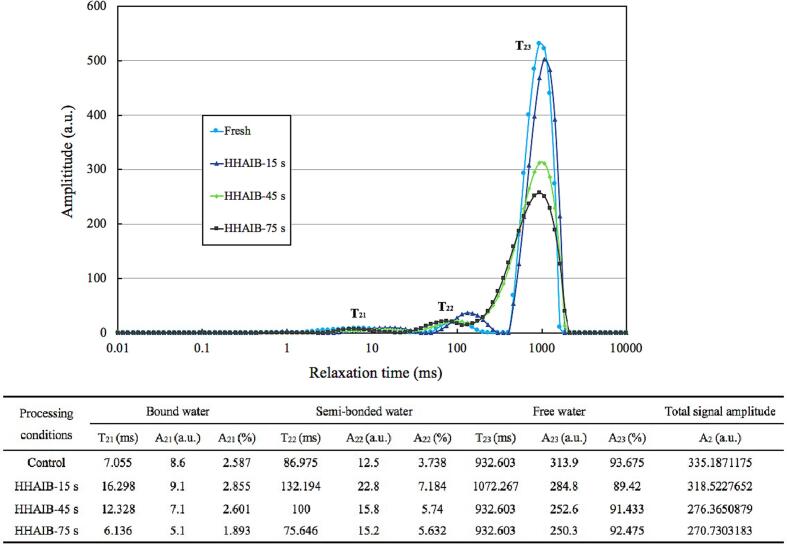
Changes in relaxation time (T_21_, T_22_, and T_23_) and the relative peak areas (A_21_, A_22_, and A_23_) of the whole tomato during HHAIB heating.

NMR was frequently applied to investigate the variation in water states and fractions in cellular compartments through the relaxation time of hydrogen protons (Liu et al., 2022b). It could also monitor the state and content of water in tomatoes after different HHAIB durations by measuring the transverse relaxation time (T_2_) and percentage of the water fraction (A_2_), as shown in Fig. 4. For raw tomato samples (control), there were three water phases in the T_2_ spectrum, namely bound water in cell walls (with the lowest T_21_ at 7.055 ms), immobilized water in the cytoplasm and extracellular space (the medium T_22_ at 86.975 ms) and free water in vacuoles (the highest T_23_ at 932.603 ms), respectively (Lagnika et al., 2013). Most of the water in tomatoes was free water in vacuoles with the longest relaxation time due to its high mobility. After HHAIB processing, the peak amplitude decreased, and the T_2_ curve first moved to the right after 15 s of processing and then moved to the left with the increase of processing time compared with the fresh sample curve. A similar phenomenon was observed by Wang et al. (2018) and Deng et al. (2019a), who found that HHAIB processing induced a reduction of peak amplitude and the T_2_ curve first shifted to the right and then to the left as the processing time increased for grapes and apricots. This phenomenon that the T_2_ curve first slightly shifted to the higher relaxation direction was probably attributed to the fact that thermal energy caused the samples to instantaneously absorb large amounts of heat, resulting in decrease in the water-holding capacity of cells, and thus a slight increase in the degree of water freedom with three different states (Shao and Li, 2013, Li et al., 2014a). With increasing HHAIB processing time, the accumulated heat in tomatoes caused the destruction of cell structure, as confirmed by SEM images (Fig. 2), increasing the chemical exchange between hydroxyl protons and water on the rigid cell wall polysaccharides and water loss from the cytosolic cell fluids and cell walls (Deng et al., 2019a). At the same time, free water in the cell evaporated, causing free water loss, and thus the T_2_ relaxation time of samples decreased.

The relative areas of T_2_ populations were labeled as A_2_, and A_21_, A_22_, and A_23_ which were introduced to represent the percentage of the three states of water, namely bound water in cell walls, immobilized water in the cytoplasm and extracellular space and free water in vacuoles, respectively (Lagnika et al., 2013, Wang et al., 2022). As shown in Fig. 4, A23 representing the percentage of free water, was the leading group (93.675 %). At the beginning of processing, HHAIB caused an increase of A_21_ and A_22_ and a slight reduction of A_23_, which could be attributed to the free water that first began to evaporate after heating because the degrees of freedom increased for all three moisture states. With the increase in the HHAIB treatment time, both A_21_ and A_22_ decreased and A_23_ increased. These findings might indicate that HHAIB heating caused the decrease of the binding capacity of the bound water in the cell wall polysaccharides or other components, the transformation of immobilized water in the cytoplasm and extracellular space into more mobile water, and the disruption of cell ultrastructure, resulting in the transfer of water and the increase of free water percentage (Deng et al., 2019a). However, the decrese in integral area of A_23_ (i.e. moisture content) indicated that the water continuously vaporized during blanching.

The changes in water states and fractions of tomatoes during the HHAIB heating process were confirmed by the NMR technique, which was another important factor to consider for a detailed explanation of the HHAIB peeling mechanism. Within the heating time of 75 s, the surface temperature rose from room temperature to above 80 °C, and the rate of surface heating first increased sharply and then decreased (Fig. 1(b)). In this process, the internal vapor pressure gradually built up, and the thin-walled cells with high water content gradually expanded and then ruptured, which resulted in a reduction in peel adhesive strength, causing a layer separation or loosening between tomato peel and flesh, thus showing a higher peeling easiness. In addition, skin cracking was also considered to be related to the increase in internal vapor pressure. Li et al. (2014a) and Wang et al. (2014) analyzed the phenomenon of tomato skin cracking caused by the infrared heating peeling process and proposed an explanation that the increase of internal steam pressure increased the stress in the skin film. Peel cracking occurred when skin membrane stress was higher than the critical rupture stress. Infrared-heated tomatoes were exposed to air, and the skin's internal and external pressure difference was more significant during infrared heating (Li et al., 2014a). Unlike infrared heating, in HHAIB peeling, the air with high temperature, high humidity, and high air velocity impacted the tomato surface in a short heating duration, so the internal and external pressure difference of the skins of HHAIB heated tomatoes might be relatively lower than that of infrared heated tomatoes. Therefore, the change of water states and fractions might be one of the main factors that affect the peel loosening in the HHAIB peeling process. However, for the peel cracking induced by HHAIB heated tomatoes, the weakening of cellular skin structure caused by the thermal effect might be the main factor, and the generation of internal steam pressure might be an additional factor.

### 3.5 Cell wall pectin fraction content changes

**Fig. 5:**
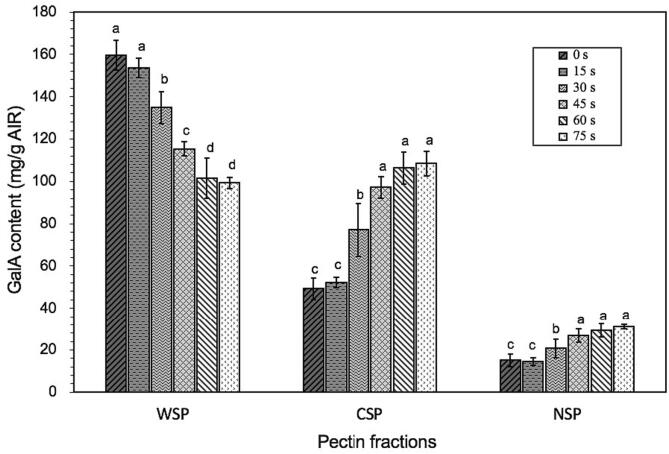
GalA contents of pectin fractions in tomato flesh during HHAIB heating.

As shown in Fig. 2(b), the degree of peel separation and the destruction degree of cell structures of the parenchymatous cells adjacent to the hypodermal cell layer gradually increased with increasing HHAIB processing time, which was assumed to be also related to the degradation of cell wall macromolecular polysaccharides between the skin layer and parenchymatous cell layer in the mesocarp caused by HHAIB induced thermal damage. Pectin is the main component supporting the cellulosic and hemicellulosic networks in the cell wall and is also the primary substance in the middle lamella that binds adjacent cells (Basanta et al., 2013, Gao et al., 2018). As the temperature rose, especially in the surface part of the tomato, leaving the internal tomato temperature mostly unchanged (Fig. 1(b)), the flesh part of tomatoes (peels and seeds) was only collected and used to measure the pectin content (WSP, CSP, and NSP) to substantiate the above hypothesis. As shown in Fig. 5, for raw samples, the amount of WSP was much higher than CSP and NSP, and the values of which were 159.66, 49.24, and 15.11 mg GalA/g AIR, respectively. The result was in agreement with the previous reports by Gao et al. (2018) that the WSP content was significantly higher than all other insoluble pectin content in tomato flesh.

During blanching, the content of pectin fractions changed significantly, among which the WSP content decreased sharply with the increase of HHAIB processing time from 0 to 75 s. This change was consistent with reports by Gao et al. (2018) in tomatoes during ultrasound post-assisted lye peeling processing and Oshima, Kato, and Imaizumi (2021) in persimmons during blanching. The WSP polymers were loosely linked to the cell wall by non-covalent and non-ionic bonds, which mainly existed in the middle lamella, with a high degree of esterification and no tight binding (Christiaens et al., 2012b, Colodel et al., 2018). The decrease of WSP might be attributed to the thermos-solubilisation of WSP at high temperatures and then leaching out of the tissue during HHAIB heating (Christiaens et al., 2012b, Deng et al., 2018). Sila et al. (2006) also reported that the WSP fraction was the most thermolabile when pectin fractions exhibited different heat sensitivity. In terms of CSP and NSP, the content of CSP significantly increased with the increase of blanching time, which was in accordance with the previous report by Oshima, Kato, and Imaizumi (2021), and the NSP content increased slightly. Thus, the decrease in WSP might be ascribed partially to the change from WSP to CSP promoted by thermal processing. The CSP was mainly linked to cell wall polysaccharides through ionic bonds (Colodel, Vriesmann, & Petkowicz, 2018). During a 75 s HHAIB heating, existing pectic substances might be challenging to achieve the inactivation of PME (a heat-resistant enzyme) under this processing condition (Christiaens et al., 2012a), and WSP might partly change to CSP by the Ca^2+^ interaction in the existence of PME activity (Oshima, Kato, & Imaizumi, 2021). Therefore, thermal energy in HHAIB processing could result in the breakdown of water-soluble pectin in the middle lamella, which reduced the adhesion between tomato peel and flesh cells and caused tissue damage, thus improving peeling easiness and peelability.

### 3.6 Mechanical changes of tomato skin

**Fig. 6:**
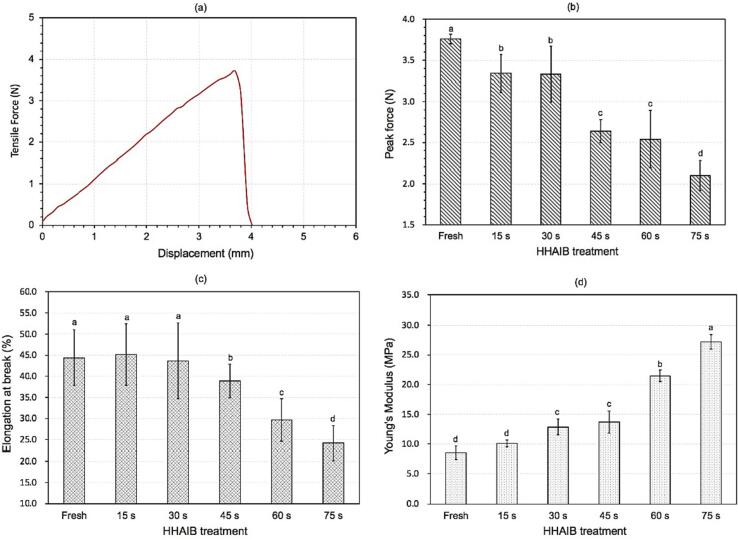
A representative force–deformation curve of peel specimen (fresh sample) (a); changes of peak force (b), elongation at break (c), and Young's Modulus (d) of peel specimen treated by different HHAIB treatments.

The mechanical properties of tomato peel were closely related to its cracking behavior (Gao et al., 2018, Vidyarthi et al., 2019b). Tomato peels with complex structures could be regarded as an elastic material, so the tensile strength, elongation values and Young's modulus were analyzed, as shown in Fig. 6. (Wang et al., 2014, Vidyarthi et al., 2019b). Results demonstrated that the tensile strength and elongation values of tomato peel were reduced and the Young's Modulus increased with the increase in HHAIB processing time, or in other words, with the surface temperature of tomatoes. These phenomena indicated that HHAIB treatment made tomato peels more challenging to stretch and more likely to crack, i.e., the treatment increased the stiffness and frangibility of peels (Li et al., 2014b). The trends were following the previous reports on the mechanical changes of tomato peels during ultrasound combined with the lye peeling process (Gao et al., 2018) and infrared peeling process (Vidyarthi et al., 2019b, Li et al., 2014b). The mechanical properties of tomato peel are closely related to its structure and composition. In the study of tomato maturation and growth, Young's modulus of tomato peel was also observed, which was attributed to the changes in cuticle structure and cellulose network polymers in fruit peel (Hetzroni, Vana, & Mizrach, 2011). The change of mechanical properties due to HHAIB heating was possibly attributed to the following reasons: (1) HHAIB heating led to the melting and redistribution of the epicuticular wax layer, resulting in fruit peel thickness reduction, which was confirmed by LSCM images (Fig. 3); (2) The large amount of heat produced by high temperature and humidity of air caused the alteration of polysaccharide matrix in tomato peel, and TFM and SEM images showed that fruit peel cells were destroyed after HHAIB heating (Fig. 2 and Fig. 3); (3) the impact of the air with high air velocity on tomato peel and internal vapor pressure underneath tomato peel after heating resulted in weakening the overall strength of skin network (Fig. 6), causing the decrease of tensile strength. Liu et al. (2022a) reported that different environmental conditions could change the elastic modulus of fruit peel, the pectin and cellulose viscosity, the enzyme activities, water content, etc. During HHAIB heating, the processing environment with high temperature, relative humidity, and air velocity altered the peel's biomechanical properties, thus affecting the stiffness and rupture strength, which might play an important role in peel cracking.

## 4 Conclusion

In the study, the peeling mechanism of tomato by HHAIB heating was investigated from multiple aspects, including the microstructure of skin, the ultrastructure of the skin layer, the water state of the whole tomato, cell wall pectin fraction content changes of tomato flesh, and the mechanical property of tomato skin. During the HHAIB peeling process, the increase in surface temperature resulted in peeling easiness and peelability, which was closely related to HHAIB-induced loosening and cracking of tomato skin. HHAIB heating resulted in the melting and redistribution of the epicuticular wax layer, structural damage on the cuticular membrane, the production of more and larger random cracks on the epidermal layer, and increased stiffness and frangibility of peels, which in turn promoted peeling cracking. In addition, the breakdown of water-soluble pectin of tomato flesh, severe degradation of the parenchymatous cells adjacent to the hypodermal cell layer, and the increase of the internal vapor pressure caused by cell fluid vaporization reduced the adhesion between tomato peel and flesh cells, resulting in peel loosening. These results increase the understanding of HHAIB peeling technology to control better the extent of peeling and avoid unnecessary losses in industrial production.

## CRediT authorship contribution statement

**Yu-Hao Zhou:** Conceptualization, Methodology, Writing – original draft. **Sriram K. Vidyarthi:** Writing – review & editing. **Parag Prakash Sutar:** Writing – review & editing. **Buer Ha:** . **Qing-Hui Wang:** Resources. **Fa-Tao He:** Resources. **Ming-Qiang Xu:** Conceptualization, Funding acquisition. **Wen-Qiang Zhang:** Methodology. **Hong-Wei Xiao:** Conceptualization, Funding acquisition.

## Declaration of competing interest

The authors declare that they have no known competing financial interests or personal relationships that could have appeared to influence the work reported in this paper.

## Data Availability

Data will be made available on request.

## References

[b0005] Ayvaz H., Santos A.M., Rodriguez-Saona L.E. (2016). Understanding tomato peelability. Comprehensive Review in Food Science and Food Safety.

[b0010] Basanta M.F., de Escalada Plá M.F., Stortz C.A., Rojas A.M. (2013). Chemical and functional properties of cell wall polymers from two cherry varieties at two developmental stages. Carbohydrate Polymers.

[b0015] Christiaens S., Buggenhout S.V., Houben K., Chaula D., Loey A.M.V., Hendrickx M.E. (2012). Unravelling process-induced pectin changes in the tomato cell wall: An integrated approach. Food Chemistry.

[b0020] Christiaens S., Mbong V.B., Buggenhout S.V., David C.C., Hofkens J., Loey A.M.V. (2012). Influence of processing on the pectin structure–function relationship in broccoli purée. Innovative Food Science & Emerging Technologies.

[b0025] Colodel C., Vriesmann L.C., Petkowicz C.L.O. (2018). Cell wall polysaccharides from Ponkan mandarin (Citrus reticulata Blanco cv. Ponkan) peel. Carbohydrate Polymers.

[b0030] Deng L.Z., Pan Z., Mujumdar A.S., Zhao J.H., Zheng Z.A., Gao Z.J. (2019). High-humidity hot air impingement blanching (HHAIB) enhances drying quality of apricots by inactivating the enzymes, reducing drying time and altering cellular structure. Food Control.

[b0035] Deng L.Z., Pan Z., Zhang Q., Liu Z.L., Zhang Y., Meng J.S. (2019). Effects of ripening stage on physicochemical properties, drying kinetics, pectin polysaccharides contents and nanostructure of apricots. Carbohydrate Polymers.

[b0040] Deng L.Z., Mujumdar A.S., Yang X.H., Wang J., Zhang Q., Zheng Z.A. (2018). High humidity hot air impingement blanching (HHAIB) enhances drying rate and softens texture of apricot via cell wall pectin polysaccharides degradation and ultrastructure modification. Food Chemistry.

[b0045] Fava J., Nieto A., Hodara K., Alzamora S.M., Castro M.A. (2017). A study on structure (micro, ultra, nano), mechanical, and color changes of solanum lycopersicum L. (cherry tomato) fruits induced by hydrogen peroxide and ultrasound. Food Bioprocess Technology.

[b0050] Floros J.D., Chinnan N.S. (1990). Diffusion phenomena during chemical (NaOH) peeling of tomatoes. Journal of Food Science.

[b0055] Floros J.D., Chinnan N.S. (1988). Microstructural changes during steam peeling of fruits and vegetables. Journal of Food Science.

[b0060] Gao R., Ye F., Lu Z., Wang J., Shen X., Zhao G. (2018). A novel two-step ultrasound post-assisted lye peeling regime for tomatoes: Reducing pollution while improving product yield and quality. Ultrasonics-Sonochemistry.

[b0065] Garcia E., Barrett D.M. (2006). Peelability and yield of processing tomatoes by steam or lye. Journal of Food Processing and Preservation.

[b0070] Garrote R.L., Silva E.R., Bertone R.A. (2000). Effect of thermal treatment on steam peeled potatoes. Journal of Food Engineering.

[b0075] Hetzroni A., Vana A., Mizrach A. (2011). Biomechanical characteristics of tomato fruit peels. Postharvest Biology and Technology.

[b0080] Lagnika C., Zhang M., Mothibe K.J. (2013). Effects of ultrasound and high pressure argon on physico-chemical properties of white mushrooms (Agaricus bisporus) during postharvest storage. Postharvest Biology and Technology.

[b0085] Li X., Pan Z., Atungulu G.G., Wood D., McHugh T. (2014). Peeling mechanism of tomato under infrared heating: Peel loosening and cracking. Journal of Food Engineering.

[b0090] Li X., Pan Z., Atungulu G.G., Zheng X., Wood D., Delwiche M. (2014). Peeling of tomatoes using novel infrared radiation heating technology. Innovative Food Science and Emerging Technologies.

[b0095] Liu H., Han X., Fadiji T., Li Z., Ni J. (2022). Prediction of the cracking susceptibility of tomato pericarp: Three-point bending simulation using an extended finite element method. Postharvest Biology and Technology.

[b0100] Liu, Y. Y., Sun, W. H., Li, B. Z., Wang, Y., Lv, W. Q., Shang, N., et al. (2022b). Dehydration characteristics and evolution of physicochemical properties of *Platycodon grandiflorum* (Jacq. A.DC.) roots (PGR) during pulse-spouted microwave vacuum drying (PSMVD). *Industrial Crops & Products, 177*, 114449.

[b0105] Liu Z., Li Z., Yue T., Diels E., Yang Y. (2020). Differences in the cell morphology and microfracture behaviour of tomato fruit (Solanum lycopersicum L.) tissues during ripening. Postharvest Biology and Technology.

[b0110] Mintz-Oron S., Mandel T., Rogachev I., Feldberg L., Lotan O., Yativ M. (2008). Gene expression and metabolism in tomato fruit surface tissues. Plant Physiology.

[b0115] Mohr W.P. (1990). The influence of fruit anatomy on ease of peeling of tomatoes for canning. International Journal of Food Science and Technology.

[b0120] Oshima T., Kato K., Imaizumi T. (2021). Effects of blanching on drying characteristics, quality, and pectin nanostructures of dried cut-persimmons. LWT.

[b0125] Redgwell R.J., Melton L.D., Brasch D.J. (1992). Cell wall dissolution in ripening kiwifruit (Actinidia deliciosa): Solubilization of the pectic polymers. Plant Physiology.

[b0130] Shao X., Li Y. (2013). Application of low-field NMR to analyze water characteristics and predict unfrozen water in blanched sweet corn. Food Bioprocess Technology.

[b0135] Shen Y., Khir R., Wood D., McHugh T.H., Pan Z.L. (2020). Pear peeling using infrared radiation heating technology. Innovative Food Science and Emerging Technologies.

[b0140] Sila D.N., Doungla E., Smout C., Van L.A., Hendrickx M. (2006). Pectin fraction interconversions: Insight into understanding texture evolution of thermally processed carrots. Journal of Agricultural & Food Chemistry.

[b0145] Toivonen P.M.A., Brummell D.A. (2008). Biochemical bases of appearance and texture changes in fresh-cut fruit and vegetables. Postharvest Biology and Technology.

[b0150] Vidyarthi S.K., El- Mashad H.M., Khir R., Zhang R., Tiwari R., Pan Z. (2019). Evaluation of selected electric infrared emitters for tomato peeling. Biosystems Engineering.

[b0155] Vidyarthi S.K., El-Masha H.M., Khir R., Zhang R., Tiwari R., Pan Z. (2019). Quasi-static mechanical properties of tomato peels produced from catalytic infrared and lye peeling. Journal of Food Engineering.

[b0160] Wang J., Fang X.M., Mujumdar A.S., Qian J.Y., Zhang Q., Yang X.H. (2017). Effect of high-humidity hot air impingement blanching (HHAIB) on drying and quality of red pepper (Capsicum annuum L.). Food Chemistry.

[b0165] Wang J., Mujumdar A.S., Deng L.Z., Gao Z.J., Xiao H.W., Raghavan G. (2018). High-humidity hot air impingement blanching alters texture, cell-wall polysaccharides, water status and distribution of seedless grape. Carbohydrate Polymers.

[b0170] Wang J., Pei Y.P., Chen C., Yang X.H., An K., Xiao H.W. (2023). High-humidity hot air impingement blanching (HHAIB) enhances drying behavior of red pepper via altering cellular structure, pectin profile and water state. Innovative Food Science and Emerging Technologies.

[b0175] Wang J., Xiao H.W., Fang X.M., Mujumdar A.S., Vidyarthi S.K., Xie L. (2022). Effect of high-humidity hot air impingement blanching and pulsed vacuum drying on phytochemicals content, antioxidant capacity, rehydration kinetics and ultrastructure of Thompson Seedless grape. Drying Technology.

[b0180] Wang Y., Li X., Sun G., Li D., Pan Z.L. (2014). A comparison of dynamic mechanical properties of processing-tomato peel as affected by hot lye and infrared radiation heating for peeling. Journal of Food Engineering.

[b0185] Zhang J.S., Chen X.J., Zielinska S., Sutar P.P., Li S.B., Deng L.Z. (2023). An improved vacuum-steam pulsed blanching machine design and garlic scape blanching verified experiment. Innovative Food Science and Emerging Technologies.

[b0190] Zhang Y., Zielinska M., Vidyarthi S.K., Zhao J.H., Pei Y.P., Li G. (2020). Pulsed pressure pickling enhances acetic acid transfer, thiosulfinates degradation, color and ultrastructure changes of “Laba” garlic. Innovative Food Science and Emerging Technologies.

[b0195] Zhou Y.H., Sutar P.P., Vidyarthi S.K., Zhang W.P., Yu X.L., Li X.Y. (2022). High-humidity hot air impingement blanching (HHAIB): An emerging technology for tomato peeling. Innovative Food Science and Emerging Technologies.

[b0200] Zhou Y.H., Vidyarthi S.K., Yang X.H., Duan X., Liu Z.L., Mujumdar A.S. (2022). Conventional and novel peeling methods for fruits and vegetables-a review. Innovative Food Science and Emerging Technologies.

