# Supplementary material for: Peeling mechanism of tomato induced by HHAIB: Microscopic, ultrastructure, chemical, physical and mechanical properties perspectives
Source: Food Chem X. 2023 Nov 22;20:101028. doi: 10.1016/j.fochx.2023.101028 (PMC10740078; doi:10.1016/j.fochx.2023.101028)
Supplement: Supplementary data 1 [file mmc1.docx]

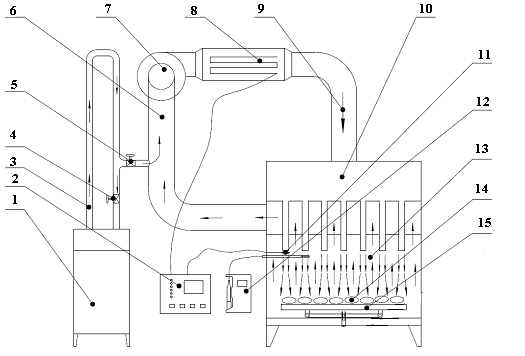


Fig. S1. Schematic diagram of equipment used for high-humidity air impingement blanching (HHAIB)

1. Superheated steam generator 2. Temperature and air velocity sensor 3. Steampipe 4. Atmospheric pressure valve 5. Superheated steam close valve 6. Recirculating air pipe 7. Centrifugal fan 8. Electric heating 9. Inlet air pipe 10. Air distribution cabinet 11. Temperature sensor 12. Humidity sensor 13. Impingement chamber with series of round nozzles 14. Materials 15. Material tray
